# Ovarian Cysts Complicated with Hernia, Hæmorrhoids, Prolapsus Vaginæ and Fibroid Tumors of the Uterus–Successful Ovariotomy

**Published:** 1880-12

**Authors:** T. P. Seeley


### ARTICLE II.

### OVARIAN CYSTS COMPLICATED WITH HERNIA, HÆMORRHOIDS, PROLAPSUS VAGINÆ AND FIBROID TUMORS OF THE UTERUS—SUCCESSFUL OVARIOTOMY. (Read before the West Side Chicago Medical Society, May 10th, 1880.) By T. P. SEELEY, A.M., M.D.

The following case, owing to its complications, doubtful diagnosis at first, and the length and variety of treatment tried, seems to justify a somewhat detailed description.

As it is stated by the patient that she has the names of 150 physicians who have visited her during the five years of her suffering, some may recognize an old acquaintance.

Being called to see the patient, Mrs. M. W., in the night of April 21st, 1876, I found her suffering extremely from abdominal pains somewhat intermittent in character.

On inspection, the abdomen was found about the size of the sixth month of pregnancy, somewhat tympanitic and tender. She was suffering also from inflamed hæmorrhoids, which, with great constipation of the bowels, had been a source of misery to her for years.

A digital examination revealed a great enlargement and prolapsus of the uterus and inflammation of the os. For immediate relief of her intense suffering, I gave a hypodermic injection of morphia and atropia, and ordered a warm stimulating enema to empty the constipated bowels.

The patient, who was forty years of age, American, of spare build, dark complexion, with a face indicating much energy of character, informed me that she had suffered from painful menstruation from childhood; had been married twice—at twenty-three and twenty-eight years of age—had one living child ten years ago and four abortions since.

In the years of 1872 and 1873 she had several attacks of fever, with severe pains in the left ovarian region, lasting one or two months.

In 1874 and 1875 she suffered very much from menorrhagia, piles and dysuria, and in January of 1876 a hernia was discovered on the right side, she having been under the treatment of several prominent homœopathic and eclectic physicians. The next day, April 22d, the bowels having been thoroughly evacuated, on examination by palpation and percussion, a firm, irregularly rounded tumor, of about the size of the fist, was found just below the umbilicus.

On a vaginal examination, the tumor, which seemed an enlargement of the uterus, was found quite hard and only slightly moveable.

On using the speculum, a small elastic catheter, with stylet, was passed five inches into the uterus, the ordinary sound being prevented from entering by a flexure of the canal. The tumor was diagnosed as a fibroid of the uterus, the diagnosis being afterwards confirmed by several other physicians.

As she objected to hypodermic injections, which were first tried, she took by mouth Squibbs' fluid extract of ergot 15 to 20 drops three times a day.

The bowels were regulated by aperients and diet, and occasional attacks of colic were relieved by warm enemas, antispasmodics, and anodynes. Under this treatment her general health was much improved and her size considerably diminished, the circumference at the umbilicus being reduced in three months from 38 to 32 inches.

During my absence at the Centennial exposition she passed into other hands for a time, but the following April returned to me, increased in size and suffering much from hæmorrhoids, constipation and dysuria. The ergot was resumed, with other suitable treatment, including the Faradaic current and a Hodge pessary to support the uterus. Temporary relief was afforded, but the tumor continued to increase, and another developed on the right side. At the time of the meeting of the American Medical Association in this city, in 1877, I invited Dr. E. Cutler, of Boston, who had read a paper giving the favorable results of galvano-puncture in a large number of cases of fibroids, to see the patient with me. As he thought the case favorable for the operation, and the patient readily consented, at my request and in the presence of Drs. Byford, Clarke, and other medical gentlemen he introduced one needle into the tumor but, finding, on a microscopic examination of the fluid, removed by the needle, characteristic ovarian corpuscles, he proceeded no farther. The puncture healed readily, and very little pain or inconvenience resulted from the operation.

As it seemed to me that we had a fibroid of the uterus, as well as an ovarian tumor or two, the continued current of electricity was tried for some time with apparent benefit, and the patient was for some time under the care of specialist electricians.

The 13th of March, 1878, I was again sent for, and found her increased in size and all her old symptoms aggravated, the left tumor being somewhat fluctuating, though still quite tense. The piles were enlarged and very painful, and there was prolapsus of the vagina, with the uterus forming a tumor of the size of the two fists projecting from the labia. Dyspnœa had gradually increased, and several times she had severe attacks of vomiting, ejecting a large quantity of dark fluid and getting temporary relief. Her bowels were irregular, and she was much troubled with colic.

On the 22d of March, with the assistance of Drs. Wm. E. Clarke and C. E. Davis, I used the aspirator, and drew off about a quart of fluid, giving temporary relief, followed in a few days by the return of all her distressing symptoms. The patient at length consented to the operation of ovariotomy, and the day was appointed for its performance; but it was postponed on account of the appearance of menstruation, which had been absent several months. At this time her attention was called to the reports in the daily papers of several cases of remarkable cures of ovarian tumors by Dr. E. W. Edwards. As she and her friends were very anxious to try the reputed cure by medicines, which I was informed by Dr. E. consists essentially of the use of large doses of iodide of potassium internally, a plaster of belladonna with iodine applied to the abdomen and the application of a saturated solution of iodine in creosote to the inner surface of the uterus after dilatation with sponge tents ; and as he promised a cure in about six weeks, I thought best to give the case entirely into his hands.

The patient was under his treatment for about fifteen months, when she again sent for me in October of 1879. She said that at first, and at times when she could take them, Dr. E.'s medicines reduced her size, but that her stomach was so weak she could not take them constantly, and this was the reason the doctor gave for not curing her. She had also had a prolonged attack of typhus fever (so called), which was another excuse for the failure of the cure.

Her symptoms were all aggravated, and she was much emaciated and weaker.

On measurement I found she had much increased in size, the circumference at the umbilicus having increased from 35 inches on April 20th, 1878, to 43½ inches October 19th, 1879, the distance from the ensiform cartilage to umbilicus, 6½ to 12 inches ; umbilicus to symphysis pubis, 6¼ to 9 inches : umbilicus to right superior spinous process, from 7½ to 13½ inches; and to the left, 9 to 14½ inches.

Despairing of all other means of relief, she was now fully resolved to have the operation, thinking that she might better die on the operating table, as Dr. Edwards kindly informed her she was sure to do if she submitted to the operation, than by slow torture.

Although she was suffering from dyspnœa and a slight bronchitis, caused either by a cold or the irritation of the tumor, it was thought unwise to delay the operation.

On the 8th of November, 1879, with the assistance of Drs. Byford, Clarke, Bartlett, Bridge, Danforth and Davis, Dr. E. W. Lee being present, the operation was performed. It occupied about an hour and a half, and was made under a continuous carbolized spray, ether being used for anæsthesia. An exploratory incision was first made midway between the umbilicus and pubis, in the linea alba, and then enlarged to about 9 inches in length, extending from about an inch above the pubis to an inch above the navel. Two large multilocular cysts were found and tapped, and 40 lbs of fluid removed by the trochar. There were extensive adhesions that were separated with the fingers, and a portion of the adherent omentum with vessels was ligated and cut away. The left and largest tumor was first lifted out, and the pedicle, about an inch and a quarter in diameter and three inches in length, was secured by passing a double braided carbolized silk ligature through the center, tying on each side and cutting them off short, the stump of the pedicle being left about an inch in length. The tumor on the right side was treated in the same manner.

Two sub-serous fibroids, about an inch in diameter and an inch and a half in length, were found attached to the fundus of the uterus, but left, as not likely to grow after the removal of both ovaries.

There was very little hæmorrhage. The cavity was carefully washed out with soft sponges, one being left in the cul-de-sac till the wound was nearly closed to absorb as much as possible of the blood and serum. The incision was closed with carbolized silk ligatures, passed with round needles from the inside, and about half an inch apart, pains being taken to have the inner surfaces of the edges of the peritoneum in contact. The last and lower stitch was taken with a common curved, lance-pointed needle, which was passed in at one side and out at the other. I mention this thus particularly, as the stitch was the only one that failed to heal readily, and there was ulceration along its course in the one lip through which the puncture was made from the outside. Nearly an inch of space was left between the last stitch and the end of the cut, so that a drainage tube might be inserted if necessary.

Whether it was the character of the puncture, or the less perfect coaptation of this portion of the wound, which prevented its healing and caused the abscess in one lip, and whether the continuous discharge which was kept up by tents contributed to or retarded the final cure, are questions of interest.

The wound was dressed and supported with adhesive straps, carbolized cotton compresses and bandages.

The two tumors, with contents, weighed forty-five pounds. The patient bore the ether and operation well and rallied favorably.

At ten p. m. there was some pain in the abdomen, increased by occasional coughing. The temperature, 100½, pulse, 90, respiration 22. About half a pint of urine was drawn with the catheter. As she was quite thirsty she was allowed to drink frequently small draughts of cold water. Gave 10 minims of Magendie's solution of morphia by hypodermic injection.

Sunday, 9th.—Patient rested well except when disturbed by cough. Temperature, 100½; pulse, 98; respiration, 24. The nurse had used the catheter at 4 a. m., and relieved her of a good quantity of urine. At 10½ the patient vomited, causing considerable pain. A hypodermic injection of 12 minims of Magendie's solution was given. As she had become somewhat accustomed to the morphia during her sickness, rather large doses were thought necessary to allay her pain. At 12½ p. m. she had nausea and vomited. Temperature, 101½; pulse, 130; respiration, 16. Has hiccoughed for a few minutes. 4½ p. m.—Cough troublesome. Temperature, 102; pulse, 120; respiration, 22. ℞. Ammonia muriat, ammoniæ carb, āā ʒj. tincturæ camph., glycerin., syrup. limonis, āā ℥j. Take a teaspoonful every three hours. 11 p. m.—Feels pretty well, but is occasionally disturbed by cough; has taken some milk porridge during the day and the catheter has been used with success every six hours. Temperature, 102; pulse, 128; respiration, 27. Hypodermic injection of 10 minims Magendie's solution. To have beef-tea and milk punch in small quantities.

November 10th, 9 a. m.—Slept quite well during the night. Temperature, 100¼; pulse, 120; respiration, 20; took beef-tea and punch several times; coughed and urinated without catheter at 3 a. m., and very freely with catheter at 8 a. m. She is quite talkative and hopeful this morning and would be very comfortable were it not for the cough. 4½ p. m.—Has taken cough mixture every three hours and has vomited mucus twice. Temperature, 100½; pulse, 112; respiration, 24. 11 p. m.—Cough troublesome, raises considerable mucus after attempts at vomiting. Temperature, 101; pulse, 112; respiration, 22.

November 11th, 10 a. m.—Patient has had much colicky pain for three hours. A warm enema of a solution of common salt being used has just secured a free movement of the bowels, with much flatus and given great relief. Temperature, 100; pulse, 108; respiration, 15. Used 10 min. Magendie's solution. 5½ p. m.—Has been quite comfortable since operation of the bowels. Temperature, 100; pulse, 104; respiration, 18. 10 p. m.—Has taken considerable beef-tea and porridge during the day. Temperature, 102; pulse, 108; respiration, 20.

November 12th, 10 a. m.—Slept most of the time till 4 a. m., then vomited and was quite restless with colicky pain till 5½, when she received a warm saline enema and passed a consistent stool and large amount of flatus. Temperature, 99½, pulse, 104; respiration, 20. 3½ p. m.—Has had considerable pain, another movement of the bowels and passed urine without catheter. Ordered Magendie's solution, 10 min. by mouth, every three hours. 11 p. m.—Quite comfortable, though she had what she calls a sinking spell on waking at 4½ p. m., felt quite faint and hands and feet were cold. Temperature, 100; pulse, 108; respiration, 18. Is to take only sufficient morphia to allay pain and secure rest. Has beef-tea and milk punch as required.

November 13th, 11 a. m.—Patient feeling quite well and hungry; took some bread and butter and tea. Temperature, 99½; pulse, 100: respiration, 16.

Dr. Bartlett coming to use the spray, she was much excited at the idea of an operation and vomited freely, the only time in the last twenty-four hours.

Dressed the wound under the spray, removing part of the stitches, union having taken place to a large extent. The wound was looking well, there being only a trace of pus. 11 p. m.—Has urinated without catheter about once in four hours; has quite severe pain. Gave hypodermic injection of 15 minims of Magendie's solution. Temperature, 99; pulse, 96; respiration, 18.

November 14th, 11 a. m.—Has rested well and is in good condition; has taken some toast, etc., and a small piece of beefsteak for breakfast. Little variation of temperature, pulse and respiration.

November 15th, 10 a. m.—Temperature, 99¼; pulse, 90; respiration, 18. Has had much colicky pain, of which she was relieved as usual, by a warm saline enema. Removed the remaining sutures without the spray. The parts are united with the exception of about an inch of the lower and superficial portion of the wound. A tent of carbolized lint was inserted for drainage and coaptation effected by compresses and rubber adhesive straps. She continued about the same, her appetite, secretions and rest being quite good, until the 19th, when she had a good deal of pain; the pulse rose to 112; temperature, 101; respiration, 17. A warm enema again secured a good movement and relief.

November 20th, 11 a. m.—Temperature, 100; pulse, 108; respiration, 18. Passed a restless and uncomfortable night. 11 p. m.—Temperature, 101½; pulse. 108; respiration, 19. Rather thin movement of the bowels, without enema; not feeling so well; seems weaker and quite despondent.

Ordered 2½ grains of quinine in capsules every 4 hours. Magendie's solution 10 min. at night.

November 21st, 11 a. m.—Rested fairly. Temperature, 101; pulse, 108; respiration, 18. Feels better. Continue quinine. 10 p. m.—Temperature, 101; pulse, 104; respiration, 19. Magendie's solution 10 min.

November 22d, 11:30 a. m.—Temperature, 101; pulse, 100; respiration, 18. Quite comfortable. Little change in the evening.

November 23d, 12 m.—Temperature, 101¼; pulse, 100; respiration, 18. Has had severe colicky pains, only partially removed by enema and movement of the bowels. Magendie's solution 10

min. was given to allay pain. 10:30 p. m.—Temperature, 102¼; pulse, 108; respiration, 18. Feverish and tossing about. Continue quinine, followed by 15 drops of tincture of chloride of iron every 4 hours; had 10 min. of Magendie's solution at 10 o'clock.

November 24th, 10 a. m., the sixteenth day after the operation.—This morning at 4 o'clock, soon after partly raising to cough and turning to lie on her right side, the patient had a large discharge of thin, dark serum of fæcal odor from the lower part of wound. On compressing the abdomen some more came out. Cleansed and dressed wound as usual.

The patient seemed much relieved by this discharge, and is feeling quite comfortable. Temperature, 98 ; pulse, 90; respiration, 17. 6:30 p. m.—Temperature, 98; pulse, 84; respiration, 18. Continued quinine and iron.

November 25th, 11 a. m.—Temperature, 97⅘; pulse, 80; respiration, 17. The patient is feeling very cheerful and the wound looking well, there being only a small discharge of pus. 11 p. m.—Temperature, 98; pulse, 72; respiration, 17. Has had a large discharge of hardened fæces.

November 26th, 12 m.—Temperature, 98; pulse, 84; respiration, 18. Feeling well. Omit morphia solution, which has been given as needed to control pain. Continue quinine and iron.

November 27th, 11 a. m.—Temperature, 98; pulse, 78; respiration, 18. Syringed abscess with warm carbolized solution, none passing into the cavity of the abdomen. Continue quinine and iron every six hours. 10:30 p. m.—Temperature, 98½; pulse, 82; respiration, 21. This afternoon, after severe pain in the rectum, several injections and hard straining she passed, with the aid of her finger, an enormous mass of hardened fæces, the largest I have ever seen, being at least two inches in diameter.

After recovering from the exhaustion of her fæcal labor she has felt much better. From the excessive hardness of this mass, I think it must have been impacted in the distended colon for months, the thin fæces having passed by the side of it.

November 28th, 11 a. m.—Temperature, 98; pulse, 80, respiration, 16. Took 10 min. Magendie's solution last night, and rested well and feels well this morning. 10 p. m.—Temperature,

$98\frac{3}{4}$; pulse, 76; respiration, 19. Appetite and secretion good. Continue tonic. The patient continued to improve, the wound gradually closing by granulation.

The hernia was quite troublesome, and required a truss. At one time it came down and became strangulated, and was with some difficulty reduced by Dr. C. E. Davis, who was called in my absence.

In about four weeks after the operation she walked into another room, and has now been doing her work for several months, though it was five months before the wound was entirely healed.

The fibroids attached to the uterus can be distinctly felt through the vagina, and she was troubled with a bearing-down sensation until I used a Hodge pessary, which gives great relief.

